# Caveolin-3 and Arrhythmias: Insights into the Molecular Mechanisms

**DOI:** 10.3390/jcm11061595

**Published:** 2022-03-14

**Authors:** Miaomiao He, Jie Qiu, Yan Wang, Yang Bai, Guangzhi Chen

**Affiliations:** Division of Cardiology, Department of Internal Medicine, Tongji Hospital, Tongji Medical College, Huazhong University of Science and Technology, 1095 Jiefang Ave., Wuhan 430030, China; hemm1026@163.com (M.H.); tjqiujie@tjh.tjmu.edu.cn (J.Q.); newswangyan@tjh.tjmu.edu.cn (Y.W.)

**Keywords:** caveolin-3, arrhythmias, ion channels, intercellular communication, metabolic perturbation

## Abstract

Caveolin-3 is a muscle-specific protein on the membrane of myocytes correlated with a variety of cardiovascular diseases. It is now clear that the caveolin-3 plays a critical role in the cardiovascular system and a significant role in cardiac protective signaling. Mutations in the gene encoding caveolin-3 cause a broad spectrum of clinical phenotypes, ranging from persistent elevations in the serum levels of creatine kinase in asymptomatic humans to cardiomyopathy. The influence of *Caveolin-3(CAV-3)* mutations on current density parallels the effect on channel trafficking. For example, mutations in the *CAV-3* gene promote ventricular arrhythmogenesis in long QT syndrome 9 by a combined decrease in the loss of the inward rectifier current (I_K1_) and gain of the late sodium current (I_Na-L_). The functional significance of the caveolin-3 has proved that caveolin-3 overexpression or knockdown contributes to the occurrence and development of arrhythmias. Caveolin-3 overexpression could lead to reduced diastolic spontaneous Ca^2+^ waves, thus leading to the abnormal L-Type calcium channel current-induced ventricular arrhythmias. Moreover, *CAV-3* knockdown resulted in a shift to more negative values in the hyperpolarization-activated cyclic nucleotide channel 4 current (I_HCN4_) activation curve and a significant decrease in I_HCN4_ whole-cell current density. Recent evidence indicates that caveolin-3 plays a significant role in adipose tissue and is related to obesity development. The role of caveolin-3 in glucose homeostasis has attracted increasing attention. This review highlights the underlining mechanisms of caveolin-3 in arrhythmia. Progress in this field may contribute to novel therapeutic approaches for patients prone to developing arrhythmia.

## 1. Introduction

The novel subcellular structures, named plasmalemmal vesicles, were first detected by Palade, G.E. et al. in 1953, and then renamed as caveolae intracellulares by Yamada, E. et al. due to their resemblance to ‘little caves’ [1]. Caveolins are the most essential proteins in caveolae, presenting in three isoforms: caveolin-1, caveolin-2, and caveolin-3. Caveolin-1 and caveolin-2 are co-expressed across many cell types, whereas caveolin-3 is specifically found in muscle tissues, such as skeletal and cardiac myocytes [2,3,4]. With the development of biochemical, cell biological, and genetic approaches, especially molecular markers, the molecular functions of caveolae have gradually been discovered, which involve the participation of homeostasis, most notably endocytosis, mechano-protection, and signal transduction. Composed of 151 amino acids, caveolin-3 has four major structural domains: the N-terminal domain, the scaffolding domain, the intramembrane domain, and the C-terminal domain [5,6,7]. Caveolin-3 exerts its effects as a scaffolding and regulatory protein for signaling molecules and moderators of ion channels and has already been linked to numerous human disease states, such as long QT syndrome, sudden infant death syndrome, myocardial hypertrophy, and diabetic cardiomyopathy [8,9,10] (Table 1). Furthermore, a study has demonstrated that the caveolin-3 protein can modify integrin function and mechanotransduction in the cardiac myocytes and intact heart; thus, modifications in caveolin-3 can result in the dysregulation of integrin function and predispose the heart to develop a myopathic phenotype [11]. Multiple ion channels are expressed in the caveolae in cardiomyocites, such as the L-Type calcium channel (LTCC), T-Type calcium channel (TTCC), voltage dependent sodium channel 1.5 (Nav1.5), the voltage-dependent K channel (Kv1.5), and inward rectifier potassium channel (Kir2.x) [12,13,14,15]. 

## 2. Caveolin-3 and Electrical Signal Propagation

### 2.1. The Sodium Current (I_Na_)

Several studies have confirmed that caveolin-3 colocalizes with the cardiac sodium channel and interacts with the dystrophin–glycoprotein complex to target multiple ion channels including *SCN5A*-encoded cardiac sodium channels (*SCN5A*, also termed Nav1.5) to the cell surface membrane [12,16,24]. *CAV-3* is a novel Long QT syndrome (LQTS)-associated gene with mutations producing a gain-of-function, LQT3-like molecular/cellular phenotype, as a pathogenic basis of sudden infant death syndrome (SIDS) [25]. It has been reported that the functional alteration of SCN5A resulting from the mutation of *CAV-3(V14L, T78M, and L79R)* is presumed to be the cause of sudden cardiac death in infants because of a significant fivefold increase in late sodium current compared with controls, just like LQT3 [16,17,26] (Table 2). Recent evidence indicates that neural nitric oxide synthase (nNOS), which mediates nitric oxide (NO) synthesis, and SCN5A form a complex with caveolin-3 in the heart, and the direct binding of caveolin-3 to nNOS suppresses the catalytic activity of nNOS [27,28]. Excessive NO synthesis and release mediated by nNOS in cardiomyocytes was shown to increase late I_Na_ [29]. It has been well documented that increased late I_Na_ caused by *CAV-3* mutation in ventricular muscle was reversed by NOS inhibitor L-NMMA, which meant that caveolin-3 was identified as an important negative regulator for cardiac late I_Na_ through a NOS-associated mechanism and contributed to both inherited arrhythmia syndromes and acquired arrhythmias in conditions [30,31]. Moreover, beta-adrenergic receptor regulation was reported to increase current densities of caveolar SCN5A through both protein kinase A (PKA)-dependent phosphorylation of sodium channels and direct G_as_ interaction with caveolin-3 which promoted the presentation of SCN5A containing caveolae to the surface membrane [32]. The roles of caveolin-3 in prolonging action potential by binding to calmodulin, which binds SCN5A and increases its slow inactivation kinetics in response to the regional increase in the concentration of calcium, have been gradually discovered. Although the above studies have confirmed the important role of caveolin-3, its specific mechanism is still unclear and may be closely related to the regulation of ion channels. 

### 2.2. The K^+^ Current (I_k_)

The inward rectifier current (I_K1_), dominating the terminal phases of cellular repolarization, maintains the resting membrane potential close to the potassium equilibrium potential and contributes to final phase 3 repolarization [33]. I_k1_ is mainly comprised of the rectifier potassium channel Kir2.1, which is one member of Kir2.x family in the human cardiac ventricle and is mainly encoded by *KCNJ2A* gene on chromosome 17 [34,35]. Kir2.x family have a unique intracellular pattern of distribution in association with specific caveolin-3 domains, which critically depends on interaction with Kir2.x- caveolin-3 binding motifs [36]. An early study demonstrated that *CAV-3* gene mutation decreased the surface expression of Kir2.1 and Kir2.2 [36,37]. Its mechanism may be closely related to the disruption in normal membrane trafficking of caveolin-3, regulating cell surface expression of Kir2.x channels, then causing decreased Kir2.x current density. Decreased Kir2.1 protein expression or function can decrease phase 3 rapid repolarization current magnitude of action potential, and prolong QT intermittent period or action potential duration, causing cardiac arrhythmia [37,38,39,40]. Moreover, a study has revealed that caveolin-3 participated in common early anterograde trafficking mechanism of the NaV1.5-Kir2.1 channelsome, which may have contributed to the potential for arrhythmias [40].

**Figure 1:**
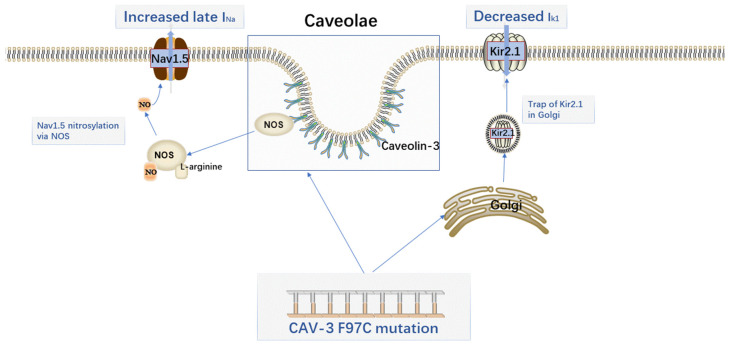
Cartoon illustration of arrhythmia mechanism caused by *Cav3* mutation (F97C). NOS, Nitric oxide synthase; NO, Nitric oxide.

The transient outward K^+^ current (I_to_), responsible for rapid initial repolarization manifested as phase 1 of the AP, was partly constituted by Kv4.2 in human ventricular myocardium. Caveolin-3 is comparably co-localized with Kv4.2 channels by co-immunoprecipitation analysis. Overwhelming evidence has suggested that *CAV-3* gene mutation results in not only slower activation and recovery of I_Kv4.2_, which could cause a reduction of I_to_ at physiological heart rates, but decreased I_Kv4.2_, which contributes to cardiac arrhythmia [31]. It has been reported that *CAV-3* gene mutation leads to dysfunction of AngII contributing to modulate a variety of ionic currents in atrial myocytes, including I_to_. In the heart, AngII binds two types of angiotensin receptors, namely, AngII receptor 1 (Ang1R) and AngII receptor 2 (Ang2R), with Ang1Rs mediating most of the AngII effects [41]. Ang1R is associated with caveolin-3 in mouse atrial myocytes that is required for the reduction in I_to_ by AngII [42]. There is increasing evidence that downstream signaling of Ang1R activation includes activation of protein kinase C (PKC) which catalyzes the hydrolysis of the membrane phospholipid, phosphatidylinositol biphosphate, producing diacylglycerol (DAG) [41,43,44,45]. Although the above studies have confirmed that the crucial role of caveolin-3 in the modulation of ionic currents, its specific mechanism is still unclear and need to be further elucidated (Figure 1). 

### 2.3. The Ca^+^ Current (I_Ca_)

Voltage-gated Ca^2+^ channels, including both the LTCC and TTCC, are the major handlers for the excitation-contraction coupling (ECC) and pacing activity of the heart [46,47,48]. TTCC have three different isoforms, CaV3.1(α1G), CaV3.2 (α1H), and CaV3.3 (α1I), which were consistently absent in human adult atrial and ventricular myocardium. In the heart, I_Ca,T_ participates in Ca^2+^ entry and Ca^2+^-dependent hormonal secretion, pacemaker activity, and arrhythmia. T-type CaV3.1 (α1G) Ca^2+^ channels play important roles in the spontaneous activity of pacemaker cells. CaV3.3 is not expressed in the heart. In the normal condition, CaV3.2 isoforms are undetectable in normal adult ventricular myocytes but are re-expressed during conditions of cardiac hypertrophy [49,50,51]. Considerable evidence indicates that the N terminus region of caveolin-3 closely interacts with CaV3.2 channels [52]. It has been reported that caveolin-3 could regulate protein kinase A modulation of the Ca(V)3.2 (alpha1H) T-type Ca^2+^ channels and attenuate cardiac hypertrophy via inhibition of T-type Ca^2+^ current modulated by protein kinase Cα in cardiomyocytes [52,53,54]. Caveolin-3 specifically inhibited the increased expression of I_Cav3.2_ and suppressed the Ca^2+^-dependent hypertrophic calcineurin-NFAT (calcineurin/nuclear factor of activated T cell) signaling pathway under pathological cardiac hypertrophy condition, thus significantly decreased the peak I_Cav3.2_ current density to improve cardiac function [53]. 

L-type Ca channels (LTCCs) plays the vital role in cardiac ECC initiated by the action potential. Recent work has shown that caveolin-3 was associated with t-tubule formation, LTCC current to the t-tubules, and localization of LTCC regulatory proteins, such as protein kinase A (PKA) and β2-adrenoceptors [15,55,56,57]. In the recently reported study, global loss of caveolin-3, rather than cardiac-specific deletion of caveolin-3 protein, resulting in the pathological loss of t-tubular I_Ca_ contributes to impaired excitation-contraction coupling and thereby cardiac function in vivo [58]. Cardiac contractile performance is mainly regulated by circulating catecholamines, which bind to β-adrenergic receptors, the main catecholamine-responsive receptors on the surface of cardiac myocytes. β-adrenergic receptors(β-ARs) have two isoforms, β1-AR and β2-ARs. Multiple studies report the clustering of β2-adrenergic receptors(β2-ARs), coupled to Gs proteins and thus, inducing cAMP production, have also been shown to co-immunoprecipitate with caveoin-3 [59]. PKA is a key mediator in the molecular mechanism of the caveolin-3 affecting LTCCs by stimulating β_2_-adrenoceptor expression [15,56].

Type 2 ryanodine receptor (RyR2), a key component of ECC in cardiomyocytes, is a cation-selective receptor channel located in the cardiac sarcoplasmic reticulum (SR) in the mammalian heart. A few Ca^2+^ ions pass through Cav1.2 to trigger a much larger Ca^2+^ release at concentration levels into the myoplasm through RyR2, thus activating action potential [60,61]. Recently, many studies have provided evidence that the RyR2 colocalized with caveolin-3 [62]. RyR2 and caveolin-3-associated dihydropyridine receptor (DHPR) forms a triple complex with HLP family which is one member of the cysteine-rich protein (Crip) [63]. The RyR2 modulates Ca^2+^ entry through caveolin-3-associated DHPR by regulating itself [15]. Caveolin-3 overexpression could lead to the reduced diastolic spontaneous Ca^2+^ waves by inhibiting the hyperphosphorylation of ryanodine receptor-2 (RyR2) at Ser2814, thus leading to the abnormal LTCC current-induced ventricular arrhythmias [64].

**Table 2:** The arrhythmia mechanism associated with change of *CAV-3* expression.

*CAV-3* Expression	Functional Alteration	Arrhythmia Implications	Related Mechanisms	Ref.
*CAV-3* mutation *(V14L, T78M*, *L79R*)	Increased late sodium current	LQT3	NOS-dependent S-nitrosylation of SCN5A	Cheng. J. et al. [30]
	Decreased Kir2.x current density	LQT9	Downstream Ang1R signaling involves the activation of PKC	Tyan. L. et al. [42]
Caveolin-3 overexpression	Reduced diastolic spontaneous Ca^2+^ waves	Ventricular arrhythmias	Inhibition of RYR2 hyperphosphorylation	Zhang. ZH. et al. [64]
*CAV-3* mutation (*S141R*)	Increased HCN4 current density	LQTS	NA	NA
Caveolin-3 downregulated expression	Activated I_Cl, swell_	Atrial fibrillation	NA	NA

NOS, Nitric oxide synthase; RYR2, Ryanodine receptor-2; Ang1R, AngII receptor 1; PKC, Protein kinase C. NA, Not Available.

Striatin (STRN) is a dynamic protein that was originally purified from highly active adenylyl cyclase rich fractions and was found to bind caveolin-3 and calmodulin (CaM) in a calcium-sensitive manner [65]. Further studies attributed the role of caveolin-3 in the interaction between STRN and CaM regulating the maturation and organization of the ECC in ventricular cardiomyocytes. The expression level of STRN was inversely proportional to the interaction of caveolin-3 with the CaM/STRN complex. Thus, caveolin-3 may mediate STRN expression by producing the opposite phenotype to silence the STRN gene, but the specific mechanism needs to be further elucidated [66].

### 2.4. The Hyperpolarization-Activated Cyclic Nucleotide Channel 4 Current (I_HCN4_)

Hyperpolarization-activated cyclic nucleotide channel 4(HCN4), also named the pacemaker channel, is the dominant isoform of the sinus node region interacting with caveolin-3 [67]. HCN4 channel function is negatively modulated by caveolin-3 to modulate the channel’s activity [68]. Of note, increased I_HCN4_ activity in the ventricle was shown to provoke ventricular automaticity resulting in ventricular arrhythmias [69]. Interestingly, LQTs-associated *CAV-3* mutations differentially modulate HCN4 channel function indicating a pathophysiological role in clinical manifestations. One study indicated that HCN4 current properties were differentially modulated by LQTS-associated *CAV-3* mutations. S141R, a *CAV-3* LQTS-associated mutation, significantly increased HCN4 current density. *CAV-3* KO resulted in a shift to more negative values in the I_HCN4_ activation curve and a significant decrease in I_HCN4_ whole-cell current density, which indicates that caveolin-3 mutations significantly accelerated the activation kinetics of HCN4 [68].

### 2.5. The Volume-Activated Cl-Channel Current (I_Cl,swell_)

Atrial fibrillation (AF) is the most common sustained cardiac arrhythmia, and it has been associated with an increased risk of stroke, heart failure, and eventually, contributing to an increased risk of cardiac and total mortality. A high level of *CAV-3* expression had a significant relationship with AF participants. Caveolin-3 concentrations in the serum samples were much higher in the group with persistent AF than the group with paroxysmal AF. Concentrations of caveolin-3 might be associated with the frequency and duration of AF [70]. AF associated with elevated chronic stretch is linked to a decrease in cardiomyocyte caveolae density and downregulation of the caveolin-3 [53,71].

Volume-activated Cl-channels are localized in the caveolae microdomains and can be activated on the mechanical stretch. Downregulation of caveolin-3 expression facilitates activation of I_Cl,swell_ and increases sensitivity to stretch 5- to 10-fold, promoting the development of AF. Caveolin-3-mediated activation of mechanosensitive I_Cl,swell_ is a critical cause of the triggering impulses that can initiate AF including AF, and this mechanism is exacerbated in the setting of chronically elevated blood pressures [72]. 

## 3. Caveolin-3 and Intercellular Communication

### 3.1. Caveolin-3 and ConnexIn 43

Gap junctions are collections of multiple intercellular channels comprised of connexons, to permit the rapid cell–cell transfer of action potentials, ensuring the coordinated contraction of the cardiomyocytes. ConnexIn 43 (Cx43) is the main component in cardiac gap junctions and is expressed in all atrial and ventricular myocytes. Reduced Cx43 expression can reduce function of gap junctions, resulting in promoting reentrant arrhythmias. There is extensive evidence that Cx43 plays a crucial role for rapid action potential transmission and signaling molecules that are associated with cardiac arrhythmias [73,74]. When the levels of full-length Cx43 protein are markedly reduced via an *M213L* mutation associated with an absence of GJA1-20k which is an auxiliary subunit for the trafficking of Cx43, the abnormal propagation of electrical impulse, including decrease in R wave amplitude, elongation of QRS complex duration, and increase in frequency of premature ventricular contractions (PVC), occurred in rats undergoing the loss of Cx43 gap junction that contributes to arrhythmias [75,76]. Oppositely, when Cx43 is activated by pinocembrin which is a flavonoid compound originated from propolis, Cx43 can be upregulated to alleviate ventricular arrhythmia in I/R rats [77]. Stem cell therapy in combination with enhanced protein expression of connexin-43 is a promising strategy against myocardial dysfunction, such as the post-infarction arrhythmias. More recently, data from the Rugowska, A et al. demonstrated that the increased expression of connexin-43 in human skeletal muscle-derived stem/progenitor cells reduced arrhythmogenic phenomena after their transplantation into the post-infarcted myocardium [78]. This is probably because that increased expression of Cx43 contributes to the reduction of inflammation and improvement of intercellular communication thus causing an indirect positive effect on calcium intracellular circulation.

Cx43 has been shown to interact with caveolin-3, and the mechanism of the relationship between Cx43 and caveolin-3 is still being explored. One study attributed a role of caveolin-1 in the regulation of Cx43. It maintains cardiac homeostasis by modulating cSrc activity. cSrc became activated to downregulate Cx43 without caveolin-1 expression. This process reduced ventricular conduction velocity and increased propensity for ventricular arrhythmias. Caveolin-3 regulating Cx43 function/expression may be a similar way as the Cx43 regulation of caveolin-1. However, more studies need to further confirm this hypothesis [79]. 

A study has shown that caveolin-3 was not only co-localized but interacts with the gap junction protein Cx43, by methods of double-hybridization and co-immunoprecipitation [80]. Moreover, the changes in caveolin-3 levels over time perfectly paralleled the pattern of changes in total Cx43 when phoneutria nigriventer spider venom (PNV) disrupt blood–brain barrier, which raise the possibility of cross-talk between Cx43 and caveolin-3 [81]. A previous study demonstrated that downregulation of caveolin-3 leads to inhibition of Cx43 gap junction communication in the lipopolysaccharide (LPS) treatment of astrocytes. Further, the specific knockout of caveolin-3 causes a downregulation of Cx43 and thus has a significant inhibitory effect on gap junctional intercellular communication (GJIC). All of those results indicated that caveolin-3 may play a crucial role in regulating Cx43 expression, but the signaling pathway between caveolin-3 and Cx43 merited further investigations [82].

### 3.2. Caveolin-3 and Dystrophin 

The dystrophin, fundamental for muscle integrity, is a critical component of the dystrophin-glycoprotein complex that acts as a connection between the extracellular matrix and intracellular cytoskeleton [83]. Mutations in the dystrophin gene contribute to dystrophinopathies, which are mostly associated with cardiomyopathies [84]. A heart lacking functional dystrophin is mechanically weak [85]. Doyle, D.D. et al. verify that dystrophin co-localizes, co-fractionates, and co-immunoprecipitates with caveolin-3, which serves to suggest directions for further research that caveolin-3 may regulate physiological functions of dystrophin as upstream regulator [86]. Recent evidence indicates that dystrophin and its associated glycoproteins are downregulated in caveolin-3 overexpression heart, causing severe cardiac tissue degeneration, fibrosis and a reduction in cardiac functions [87]. Caveolin-3 may regulate the normal processing or stoichiometry of the dystrophin complex at the protein level [88]. 

### 3.3. Caveolin-3 and Adiponectin Receptor

Adiponectin (APN) is a benign adipokine secreted cytokine with reduced expression in obesity and diabetes [89]. The primary function of APN is to increase insulin sensitivity by sensitizing the insulin receptor signaling pathway. Adiponectin receptors are G protein-coupled receptors (GPCRs) and two receptor subtypes: AdipoR1 and AdipoR2. In both AdipoR1 and AdipoR2, the N-terminal domain exists in intracellular space and C-terminal domain presents in the external region of cells. Many studies have indicated that AdipoR1 colocalized with caveolin-3, forming AdipoR1/ caveolin-3 complex via specific caveolin-3 scaffolding domain binding motifs [90]. By interacting with AdipoR1, caveolin-3 corrals downstream molecules in close proximity with AdipoR1, thus enabling proper transmembrane signaling and cardioprotection. Recently, many studies have provided evidence that high fat diet-induced diabetes disrupted the expression of *CAV-3*, which deranged eNOS signaling in diabetic myocardium and then diminished the cardioprotective effects of APN [91,92,93,94]. Overall, caveolin-3 plays an essential role in APN-induced ceramidase recruitment and activation, although the involved detailed molecular mechanisms are still unknown. 

## 4. Caveolin-3 and Metabolic Perturbation

### 4.1. Caveolin-3 and Insulin Resistance

Insulin resistance is the important defect in the pathophysiology of type 2 diabetes (T2DM). A major complication of diabetes is diabetic cardiomyopathy. The ion channel remodeling has been reported in the animal models of diabetic cardiomyopathy which could lead to a variety of arrhythmias, such as AF [95,96].

In recent years, the role of caveolin-3 in glucose homeostasis has attracted increasing attention. A previous study demonstrated that a variety of mutations were present in the *CAV-3* gene among 1–1000 patients with T2DM. A previous assessment of patients with newly developed T2DM demonstrated that K15N muta tion located in the N-terminus of caveolin-3 may lead to changes in caveolin-3 secondary structure, thus causing decreased recombinant caveolin-3 expression [97]. It has been found that decreased expression and reduced localization of caveolin-3 by *CAV-3-P104L* mutation inhibited glucose uptake and glycogen synthesis [98]. The IR/PI3K/AKT/GLUT signaling pathway has been demonstrated to be a major mechanism in the development of insulin resistance [99,100]. Insulin receptor (IR) and GLUT-4 are associated with glucose metabolism, with their expression being regulated by caveolin-3 on the cell membrane. GLUT4 is one of the most important glucose transporters and is responsible for regulating and transporting 50% to 80% of body glucose. Caveolin-3 can enhance the expression of IR by stimulating IR kinase activity and activating PI3K/AKT signaling pathway. Activated AKT promoted the translocation of GLUT-4 to the plasma membrane and enhanced glucose uptake. There is evidence that increased *CAV-3* expression contributed to GLUT4 translocation and thus ameliorated high-fat-diet (HFD)-induced glucose intolerance and insulin resistance, which indicated a positive correlation between the alternation of glucose metabolism and the level of caveolin-3 [93,101]. Conversely, decreased expression levels of *CAV-3* were observed in diabetes animal models [102]. Decreased *CAV-3* expression inhibited Akt phosphorylation, and thus protein expression of GLUT4 which are molecules downstream of Akt, was significantly decreased [98]. Moreover, caveolin-3 may play a pivotal role in 17β-estradiol (E2) actions on glucose metabolism, and there is evidence that E2 intervention reduces the incidence of diabetes [92,103]. Diabetes could cause inhibition of *CAV-3* expression by regulating the activities of various enzymes and ion channels. Those changes lead to insulin resistance, inhibition of Cx43 gap junction communication and modulation of ion channel function, which contribute to susceptibility to arrhythmias.

### 4.2. Caveolin-3 and Adiposity

Obesity is a major concern because it is a risk factor for metabolic diseases that increase mortality rates. Recent evidence indicates that caveolin-3 plays a significant role in adipose tissue and is related to obesity development. A study has demonstrated that *CAV-3*-knockout mice increased adiposity [104]. Obesity is a complex multifactorial condition that contributes significantly to cardiovascular risk including AF. In animal models with adipose tissue, it has been found that obesity is also associated with a modest increase in QTc and QT dispersion [105,106]. Moreover, high-fat-diet-fed animals showed metabolic alterations, obesity, and insulin resistance along with the induced expression of muscle-specific caveolin-3 in retroperitoneal adipocytes and skeletal muscle in the initial phase. The mechanism was partly explained by the fact that continued exposure to the high-fat diet in the initial phase produces an increase in circulating glucose and insulin levels which could induce *CAV-3* expression [107,108]. Indeed, previous studies have reported that animals fed on a high fat diet show increased oxidative stress in muscle tissue, which also could be partly explained by the induced expression of *CAV-3* [109]. In a late phase, insulin resistance becomes apparent, accompanied by an impairment of caveolin-3 levels in skeletal muscle. 

## 5. Perspective

Since it was discovered approximately 60 years ago, important functional roles and biochemical properties for caveolae have been identified. It is now clear that the caveolin-3 plays a critical role in the cardiovascular system and a significant role in cardiac protective signaling. *CAV-3* mutations have been linked to the LQT9, and the cause of underlying action potential duration prolongation is incompletely understood. However, there are numerous difficulties to be overcome in the process from basic laboratory research to clinical application. Caveolin-3 dysfunctions have been responsible for inherited arrhythmias. The importance of caveolin-3 changes in arrhythmias and downstream microdomain dysregulation may have important implications for arrhythmia generation.

Understanding the composition and functional roles of caveolin-3 in the heart as well as their contribution to arrhythmia syndromes is only the beginning. Many critical questions remain to be answered. Further basic science research and eventual randomized clinical trials are needed to define the precise mechanisms and therapeutic potential of caveolin-3 in patients with arrhythmias.

## Figures and Tables

**Table 1:** Pathogenic *CAV3* mutation associated with cardiovascular diseases.

Phenotype	*CAV3* Mutation	Serum CK Concentrations	Ref.
LQTS	*p.A85T*	NA	Vatta, M. et al. [16]
Sudden infant death syndrome	*p.V14 L*	NA	Cronk, L.B. et al. [17]
Dilated cardiomyopathy	*p.A46V*	High	Catteruccia, M. et al. [18]
Dilated cardiomyopathy	*p.T78M*	High	Traverso, M. et al. [19]
Hypertrophic cardiomyopathy	*p.T63S*	Normal	Hayashi, T. et al. [20]
Hypertrophic cardiomyopathy	*P104L*	NA	Ohsawa, Y. et al. [21]
Hypercholesterolemia	*p. Val44Met*	High	Bruno, G. et al. [22]
Atrial standstill	*p. Leu84Pro*	NA	Gal, D. B. et al. [23]

NA, Not Available; LQTS, Long QT syndrome.

## Data Availability

Not applicable.
